# A similarity-guided segmentation model for garbage detection under road scene

**DOI:** 10.1007/s12200-022-00004-9

**Published:** 2022-05-12

**Authors:** Caiyun Zheng, Danhua Cao, Cheng Hu

**Affiliations:** School of Optical and Electronic Information, Huazhong University of Science and Technology, Wuhan, 430074 China

**Keywords:** Machine vision, Semantic segmentation, Garbage segmentation

## Abstract

The development of computer vision technology provides a possible path for realizing intelligent control of road sweepers to reduce energy waste in urban street cleaning work. For garbage segmentation of seven categories under road scene, we introduce an efficient deep-learning-based method. Our model follows a lightweight structure with a feature pyramid attention (FPA) module employed in the decoder to enhance feature integration at multi-levels. Besides, a similarity guidance (SG) module is added to the decoder branches, which calculates the cosine distance between learned prototypes and feature maps to guide the segmentation results from a metric learning perspective. Our model has less than 3 M parameters and can run at over 65 FPS in an RTX 2070 GPU. Experimental results demonstrate that our method can yield competitive results in terms of speed and accuracy trade-off, with overall mean intersection-over-union (mIoU) reaching 0.87 and 0.67, respectively, on two garbage data sets we built. Besides, our model can perform acceptable category-balanced segmentation from less than 20 annotated images per category by introducing the SG module.

**Graphical Abstract:**

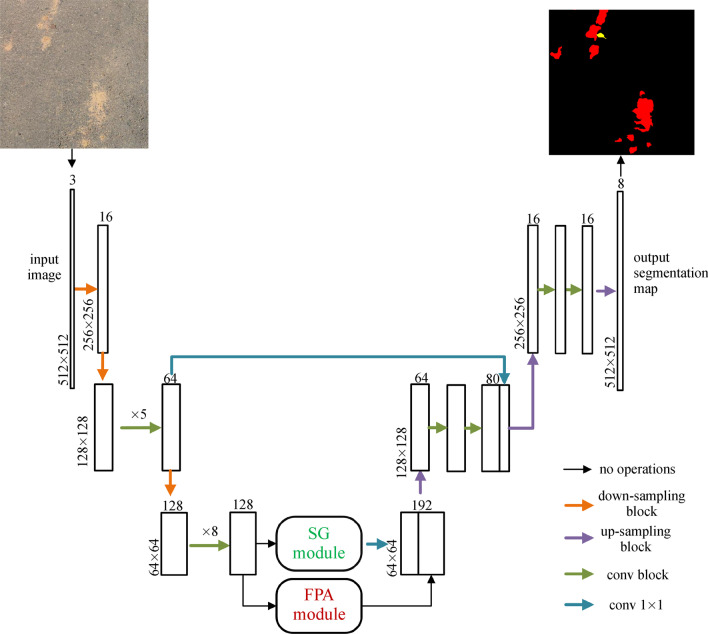

## Introduction

Road sweeper vehicles have been widely used in urban street cleaning. It has been pointed out that realizing the intelligent control of road sweepers is necessary to reduce energy waste [1]. Vision analysis is used in the intelligent control system to evaluate the cleanliness of the road surface and the density of garbage, working as the basis of adjusting the cleaning mode of road-sweepers to reduce the energy waste and improve the efficiency of the road-sweepers. Intelligent control of road sweepers involves integrated automation technology and modern computer vision technology. Recent progress on computer vision technology has shown extensive potential for real-time garbage segmentation under road scene, which plays an important part in the vision analysis of road-sweepers.

It can be challenging for practitioners to decide which method is best suited to this task. Most researchers adopted the method of transfer learning by adapting mature deep-learning-based solutions of other computer vision fields to the task of garbage segmentation by fully exploiting the powerful capability of feature extraction of convolutional neural networks (CNN) [2–8]. One key limitation in applying these methods in a practical situation is that they cannot ensure efficiency and accuracy in the meantime. Some proposed methods followed a simple and lightweight structure, GarbNet [3], for example, can only coarsely segment garbage regions of large piles, facilitating its transplant in smartphones at the cost of failure in capturing finer objects. In comparison, some other methods concentrated efforts on ensuring a high overall accuracy or specializing in tiny objects by employing time-costing multi-stage pipelines [4–6] and adopting inefficient models, such as Mask-RCNN [7] and Faster-RCNN [8]. Considering the speed and accuracy trade-offs and that the pixel-wise prediction of semantic segmentation is the best-suited form of coverage and density measurement for common garbage categories such as mud and piles of leaves under road scene, we chose to develop a real-time semantic segmentation model for this task.

This paper discusses two main difficulties in this task, namely the speed and accuracy trade-off for the segmentation task and extensive demand for a large number of pixel-wise annotated images.

**Fig. 1:**
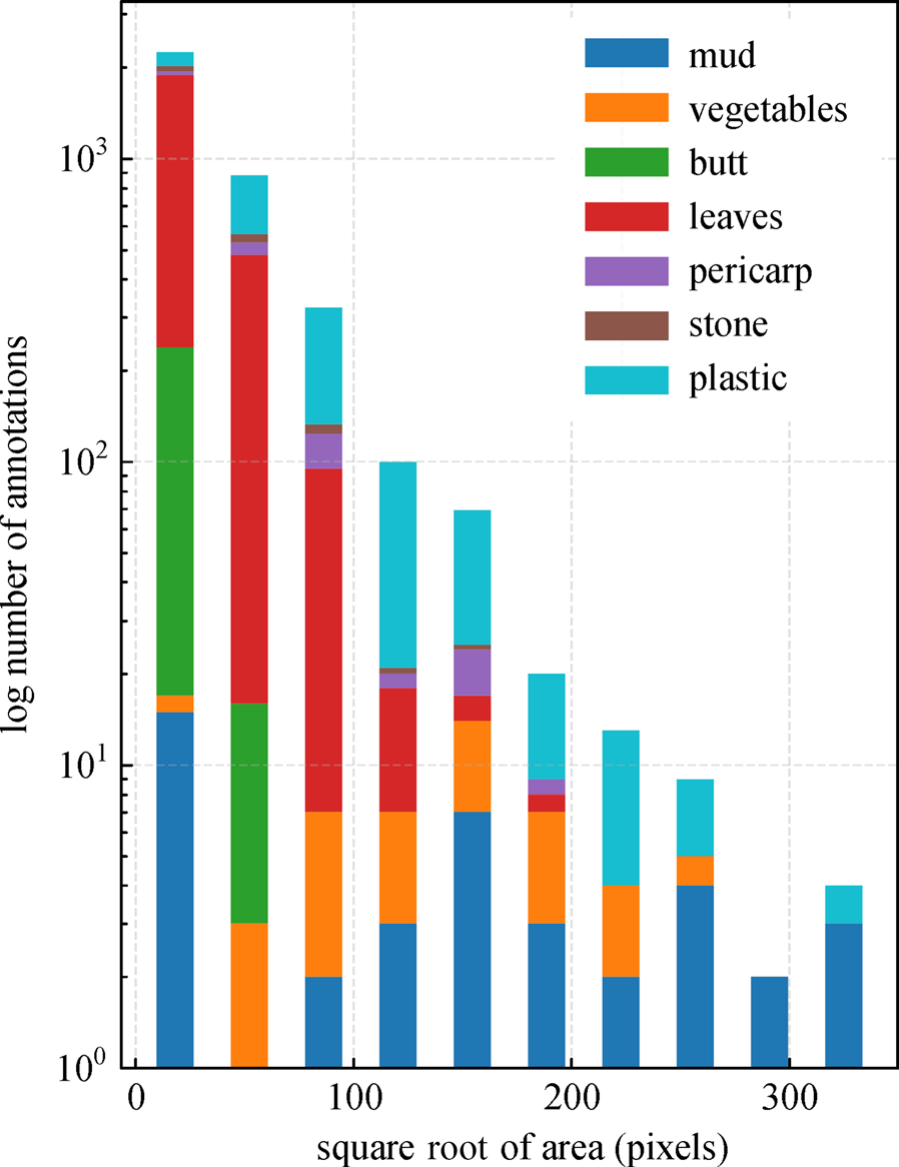
Size variations of garbage categories in our data set

The first difficulty is mainly due to the contradiction between the difficulty of garbage detection and the real-time requirements of the task. We target data sets of seven categories of garbage, including mud, vegetables, cigarette butt, leaves, pericarp, stone, and plastic, with great diversity in shapes and scales. Figure 1 demonstrates the size variability of different garbage categories in our data set. We can see that most of the butts have an area less than 50 × 50 pixels, while plastic garbage has a relatively wide range of size distribution. Various shapes and scales of garbage can be problematic. Besides, for the task of garbage segmentation under road scene rather than a blank background, not only should we take into account the diversity of garbage, but we also need to be aware of the imbalance issues between garbage foreground and road background. Therefore, how to effectively extract and integrate features at multi-scales within computation budgets, is an important issue in the design of the segmentation-model and training strategy of the model. Existing solutions for tackling the problem mentioned above mainly include improvements on model structure [9–15] and optimized training strategies [16–21]. We consider both directions in our proposed method.

The other one lies in applying segmentation model to practical use since the model’s performance relies on a large number of dense-annotated samples with diversity for training. Due to the high cost of acquiring pixel-level annotations by manual labeling, it is thus of great interest for the model to learn to perform segmentation from a limited amount of labeled samples for each category. The metric learning based method is one of the primary trends for addressing the problem. In particular, Snell et al. [22] proposed a prototypical network in which each category is represented with one specific prototype feature vector. Our model follows this idea by adopting a similarity guidance (SG) module in the decoder to tune the segmentation process. Unlike the method above targeting at few-shot learning, we regard the similarity calculated features merely as a supplement of regular segmentation. Thus, the prototypes are used to tune the segmentation rather than obtain segmentation directly from metric learning. Such structure design has the advantage of fast generalization to unseen categories and scenes with only a limited amount of annotated images, facilitating model landing in practical scenarios, at the expense of increasing little computation cost since the similarity guidance module introduces no extra learnable parameters.

In this paper, we proposed an efficient model for real-time garbage segmentation under the road scene. The contributions of this paper are as follows.

**Fig. 2:**
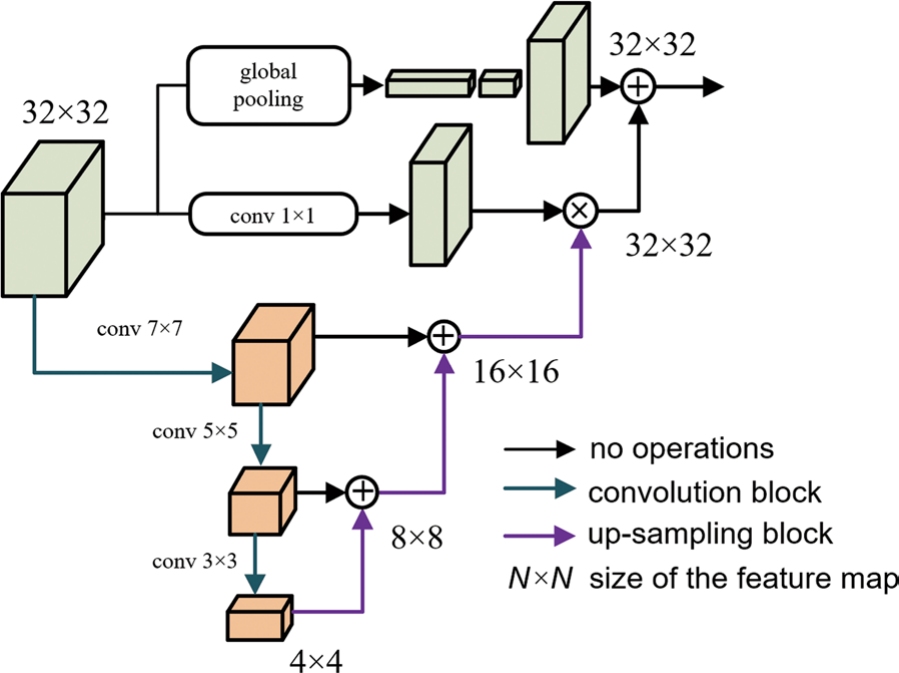
Depiction of the feature pyramid attention (FPA) module

1. We develop a real-time semantic segmentation model for the garbage segmentation task considering speed and accuracy trade-offs in practical application and the best-suited form for coverage and density measurement for common garbage categories.
2. We introduce a SG module to facilitate the model of fast generalization to unseen categories with only a limited number of annotated images, simplifying model training in practical scenarios.
3. We adopt the feature pyramid attention (FPA) module and a long-range skip connection between the encoder and the decoder to enhance the feature extraction at multi-scales and feature fusion. Besides, the compound loss function is employed for training to boost accuracy without increasing inference time. Figure 2 demonstrates the structure of the feature pyramid attention (FPA) module.

## Related work

### Architectural improvements for segmentation tasks

Considerable attention has been paid to multi-scale feature extraction and integration of segmentation models. Typical designs are mostly attributed to novel convolutional kernels (dilated convolution [9, 10]), modifications on structure (encoder-decoder structure [11], long-range skip connections [12]), and new types of plugin modules (spatial pyramid pooling (SPP) [13], atrous spatial pyramid pooling (ASPP) [14], attention mechanisms [15], etc.). In particular, we follow the same encoder arrangement as ERFNet [10], an efficient architecture for real-time semantic segmentation. In addition to adopting dilated convolutions, the novelty of ERFNet lies in the use of factorized convolutions (convolutions with 1D kernels) in convolutional blocks, which significantly reduce the computation cost while retaining a similar accuracy [10].

Some other researchers did not make progress on the architecture or layers of the model; instead, they emphasized various types of functional components that can be plugged into the model and encode multi-scale contextual information. Zhao et al. [13] and Chen et al. [14] both proposed accessory modules based on spatial pyramid pooling, while Li et al. [15] tackled the problem from the perspective of attention mechanism by specially designing a FPA module for local and global context extraction and fusion. The structure of the FPA module proposed in Ref. [15] is demonstrated as follows.

### Improvements on loss functions

Since the model is trained offline, many researchers took this advantage and paid attention to those promoting strategies that only give rise in training cost to tackle the imbalance issue arising from the intuitive gap in general segmentation tasks between foreground and background. The most common method is reweighting the cross-entropy loss [16] to prevent the model’s output from being dominated by the head category, which occupies most of the pixels in the train set. Apart from cross-entropy loss which evaluates each pixel individually and equally, loss functions based on the Dice coefficient [17–19] are proposed to tackle the extreme imbalanced situation in the task of medical image segmentation, where the target usually represents only a very small fraction of the full image. Dice coefficient [17] is a measure of overlap between two areas, equivalent to calculating the F1 score over the area. Further, Salehi et al. [19] proposed Tversky loss based on Dice loss to achieve a trade-off between precision and recall.

Shrivastava et al. [20] proposed an online hard example mining (OHEM) training strategy, considering improving training from another perspective. The motivation of the OHEM strategy is to select hard samples to boost accuracy. It is often the case in the training stage that easy samples, which make less contribution to backpropagation, usually occupy an overwhelming percent of the data sets, while the hard samples, which obtain a higher loss and contribute more to the gradient, only take a small part of the data sets. Then Wu et al. [21] made an extension of OHEM strategy to the field of semantic segmentation. For region-based object detectors, hard samples are selected region-of-interests (RoIs), which the current network performs worst. When it comes to the segmentation task, instead of RoIs selected as a whole, pixels with prediction probabilities below a specific threshold are counted as hard pixels in calculating loss functions.

### Prototype network

**Fig. 3:**
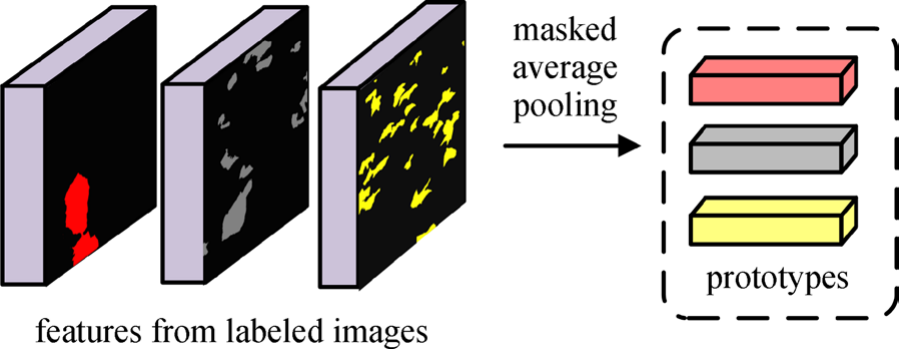
Operation of masked average pooling

There are many researches focusing on developing segmentation models trained with a limited amount of labeled samples, mostly based on the theory of metric learning [22–24]. Snell et al. [22] proposed a prototype network to generate prototype representations for each category in feature embedding space, realizing an original method for few-shot image classification. Zhang et al. [23] proposed masked average pooling and realized a SG method for segmentation task, called SG-one network, which targeted at one-shot segmentation of unseen category, and thus was often used for video tracking of one single object in a practical situation. Wang et al. [24] proposed PANet as an extension of the prototype network to multi-category segmentation tasks. The output of PANet is directly obtained by similarity calculation of metric learning, which made PANet a simple and effective design specialized for dealing with the few-shot situation. In the SG method, the feature maps of specific categories could be extracted separately by adopting masked average pooling in which only the pixels belonging to corresponding categories would be taken into account, as shown in Fig. 3.

After obtaining the prototype of each category, segmentation results can be performed by matching each pixel of the test set to the learned prototype from similarity metrics. Snell et al. [22] applied squared Euclidean distance as similarity metrics in the prototype network, while both Zhang et al. [23] and Wang et al. [24] adopted cosine distance.

## Method

In this section, we introduce our real-time proposal for garbage segmentation under road scene in detail.

### Overall framework

**Fig. 4:**
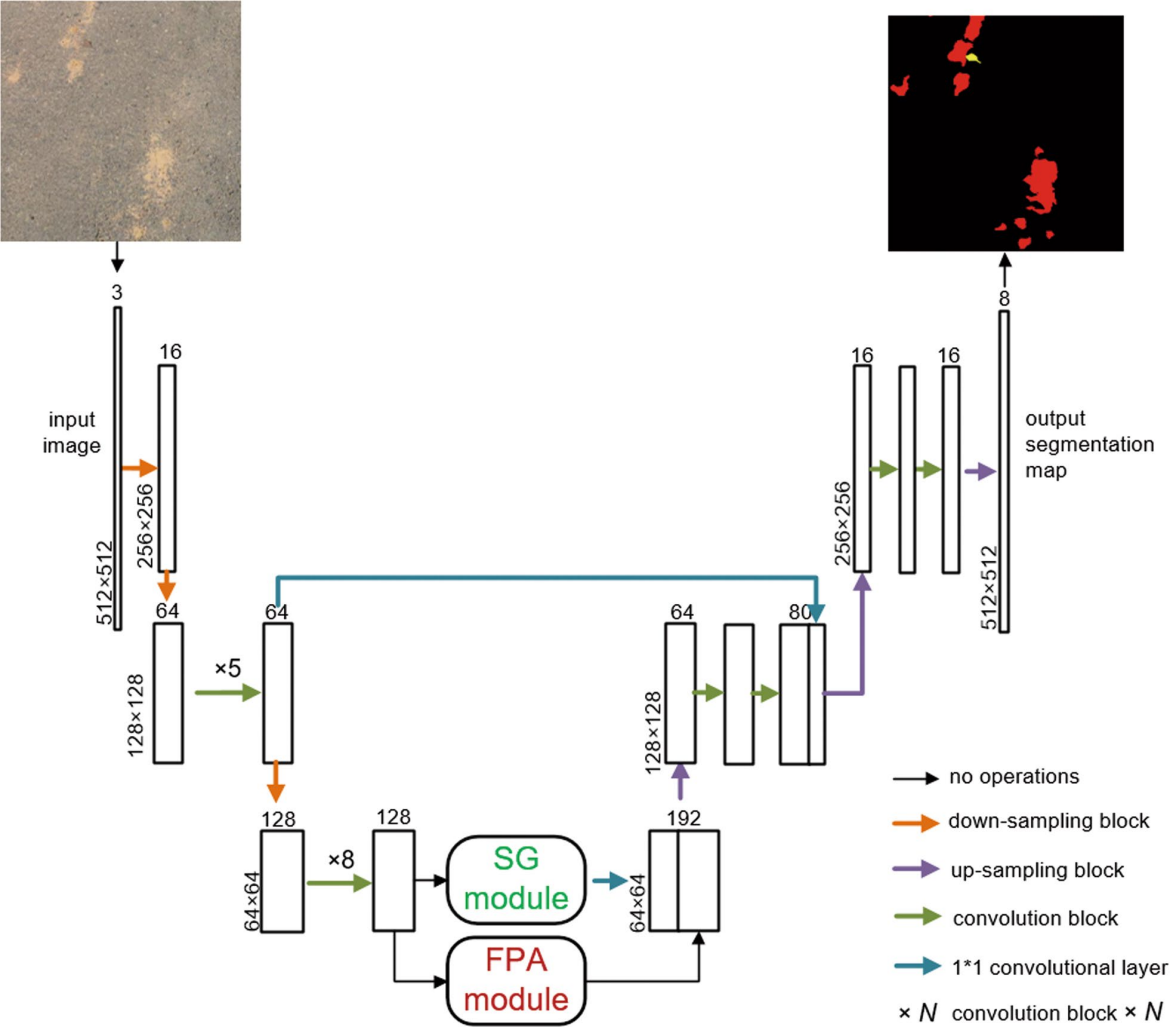
Overall framework of the proposed method

The overall framework of our proposed method is shown in Fig. 4. Our model employs an encoder-decoder structure like SegNet [11], where feature extraction of input images is implemented by residual convolutional blocks in the encoder part. At the same time, pixel-wise segmentation is produced by gradually recovering the spatial size of feature maps in the decoder part. Apart from residual convolutional blocks, down-sampling, and up-sampling blocks, we also adopt two accessory modules, FPA (feature pyramid attention) and SG (similarity guidance), in decoder structure. The former can encode contextual information at multi-scales and enhance feature fusion at multi-scales by performing pixel-level attention for high-level features, while the latter can provide similarity information to guide the segmentation process by mapping features to learned prototypes. Feature maps extracted by the encoder part are sent to the FPA and SG modules, respectively, then the output feature maps of the two modules are concatenated and passed through the rest of the convolution blocks to generate the final result. Besides, we introduce low-level features from shallow layers of the encoder into correspondingly feature maps of the decoder by long-range skip connections between the encoder and the decoder to refine the output segmentation.

### Convolutional blocks

To balance the performance and efficiency of the encoder, we follow the same strategy as ERFNet [10] in employing factorized convolutions in all basic residual convolutional blocks, and applying dilated convolutions at multiple scales in the encoder to enlarge the receptive field of feature maps.

**Fig. 5:**
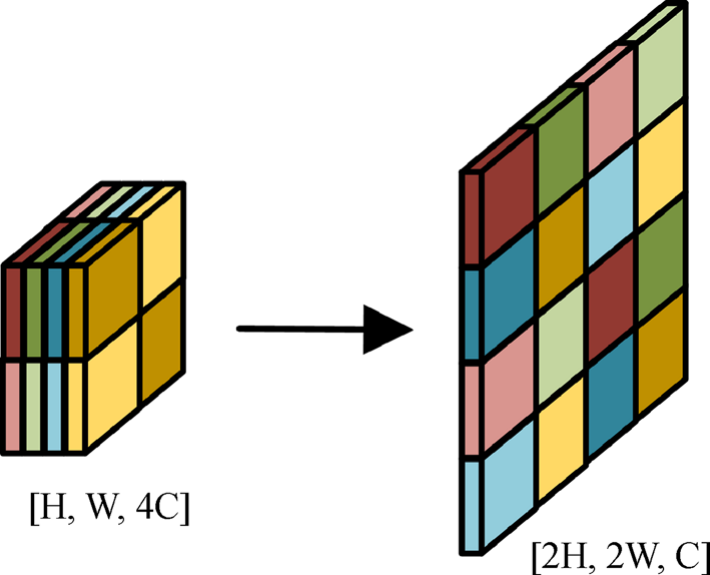
Subpixel deconvolution

For the up-sampling blocks, we do not apply max-unpooling operation like SegNet [11] and ENet [25], nor use bilinear interpolation like PSPNet [13] and DeepLab [14]. Instead, our up-sampling blocks consist of subpixel deconvolution layers [26], which is also called depth-to-space deconvolution and has been effectively applied in ExfuseNet [27] for segmentation. In contrast to conventional deconvolution operation adopted by FCN [16] and ERFNet [10], subpixel deconvolution rearranges the channels into spatial domain instead of interleaving feature maps with 0 s, as demonstrated in Fig. 5, which is reported to alleviate the alignment artifacts caused by deconvolution operation and decreases computation cost in the meantime [27].

In this paper, we build two data sets, RdIC (road in campus) and RdOC (road outside campus), to assess the performance of our model, which are detailed in Sect. 4. The effect of subpixel deconvolution and two conventional up-sampling methods (bilinear interpolation and transposed convolution) are evaluated on the two data sets, as listed in Table 1. Experiments show that applying subpixel deconvolution instead of transposed convolution in the structure of the ERFNet model as the up-sampling layer can reduce the number of model parameters by 4% and decrease the inference time by 9% while maintaining the prediction accuracy of the model; therefore we adopt the subpixel deconvolution in our proposal.

**Table 1:** Comparison results for up-sampling methods based on ERFNet

Up-sampling methods	Model complexity		mIoU	
	Params/M	Time/ms	RdIC	RdOC
Transposed convolution	2.06	13.8	0.7623	0.6304
Bilinear interpolation	1.99	14.5	0.7531	0.6130
Subpixel deconvolution	1.98	12.5	0.7578	0.6343

### Feature pyramid attention module

In contrast to sequential decoder architecture adopted by ERFNet [10], we additionally employ an FPA module for feature fusion using spatial-wise attention and long-range skip connection between the encoder and the decoder, inspired by U-Net [12], to obtain a precise output. Unlike the FPA structure proposed in Ref. [15], the adopted FPA only consists of sequences factorized 3 × 3 convolutions, namely 3 × 1 and 1 × 3 1D convolutions, instead of large kernels of 5 × 5 and 7 × 7 convolutions, respectively in pyramid structure in consideration of reducing the computation cost while retaining the prediction accuracy.

In addition, we replace the residual summation connection of FPA with concatenation for empirical accuracy gain. It has been pointed out that concatenation operation would preserve the feature maps’ feed-forward nature better than summation operation [28]. Also, we adopt concatenation for feature fusion in long-range skip connection between the encoder and the decoder.

### Similarity guidance (SG) module

We propose introducing an SG module in the decoder part to facilitate performing segmentation from only a few annotated training samples and advance the model in producing category-balanced results. The formation of the SG module mainly consists of masked average pooling operation and cosine distance calculation. The masked average pooling is only used at the training stage to generate and update the prototype vector for each category, while at the inference stage, we use the prototype vectors and the cosine distance calculation to generate results, as is demonstrated in Fig. 4. The representation feature of category *c* extracted from the $i_{{{\text{th}}}}$ sample by masked average pooling can be written as [24]

1 $$p_{i,c} = \frac{{\sum\nolimits_{x,y} {F_{i,x,y} \cdot {\text{I}\! \text{I}}\left[ {M_{i,x,y} = c} \right]} }}{{\sum\nolimits_{x,y} {{\text{I}\! \text{I}}\left[ {M_{i,x,y} = c} \right]} }},$$

where *M* denotes segmentation mask of the $i_{{{\text{th}}}}$ sample, *F* denotes feature map, and ${\mathbf{\mathbb{I}}}\left[ \cdot \right]$ is an indicator function, outputting value 1 if the argument is true or 0 otherwise.

Then similarity between the prototype vector of category *c* and pixels at all positions of the test image can be calculated as [24]

2 $$s_{x,y} = \frac{{p_{c}*F_{x,y} }}{{\left\| {p_{c} } \right\|_{2} \left\| {F_{x,y} } \right\|_{2} }},$$

where $s_{x,y}$ is the similarity value of category *c* at the pixel (*x*,*y*) of the test image, $p_{c}$ is the prototype vector for category *c*, $F_{x,y}$ is the feature vector of test image at the position (*x*,*y*), and “$*$” stands for vector multiplication.

Our SG module is inspired by few-shot learning proposals [22, 23], but we take advantage of the prototypes to learn some unique representations from the perspective of metric learning as supplementary information for conventional feature extraction. It is available for few-shot situations to directly generate the final result from similarity calculation; however, as for our task, the semantic information of the input image is not utilized effectively when more samples are provided. In our model, the SG module is used to guide the segmentation process rather than directly generate the segmentation result from the similarity value to further improve the generalization of our model in dealing with either inadequate or abundant samples.

Unlike the proposal in Ref. [24] that generates a prototype feature vector for each category based on the current batch of support images, we instead calculate an overall mean of the embedding features mapped from all training samples to represent the overall prototypes of categories. In the practical training stage, we update the prototype in each run. Apart from regular segmentation loss, we also calculate a similarity-guided loss as auxiliary optimization function.

**Fig. 6:**
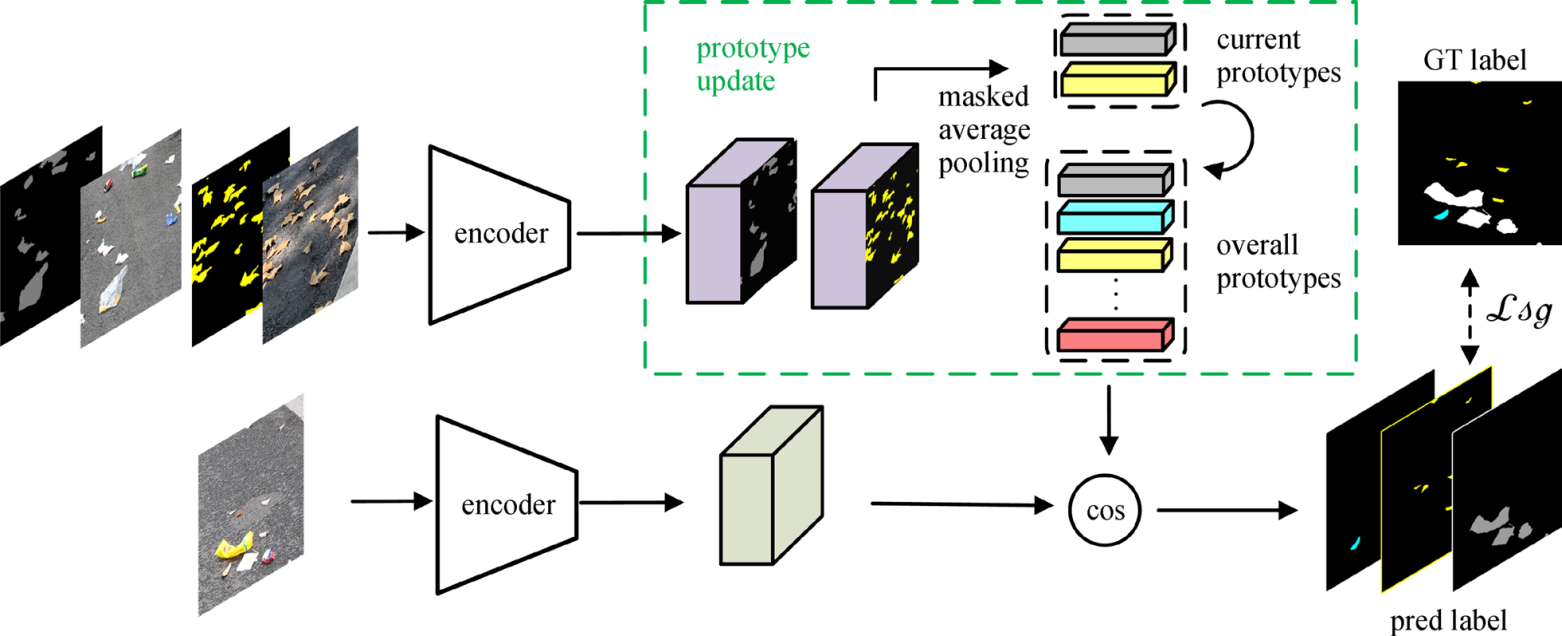
Prototype updating and calculation of similarity-guided loss

The pipeline of prototype updating and similarity-guided loss calculation is illustrated in Fig. 6. The top line of Fig. 6 illustrates prototype updating in the training stage, and the bottom line demonstrates how the SG module generates similarity results using the generated prototypes and shows the calculation of similarity-guided loss. It is worth mentioning that the similarity calculation is based on the updated prototypes generated in the last iteration; we perform the loss first then update the overall prototypes in each training iteration. To be specific, we calculate an overall mean of the embedding features mapped from all training samples to represent the overall prototypes of categories. In the block of prototype update, the overall prototypes are updated iteratively by current prototypes obtained by applying masked average pooling to the deep features of training. Loss 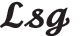 is computed between the ground truth label and intermediate output of the SG module obtained via computing the cosine distance between the prototype vectors and the feature maps at each position.

### Loss function

The model is trained end-to-end with an overall optimization 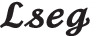 and accessory optimization 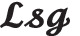, where the former is computed between the ground truth label and the final segmentation output of the model, and the latter is calculated on the ground truth and the intermediate output of SG module.


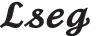
          and 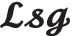 are compound losses, consisting of a weighted cross-entropy loss as the base form and a loss calculated with OHEM strategy. As for weight calculation, we adopt a general form defined as the inverse of the frequency of each category [14], which can be written as

3 $$W_{{{\text{category}}}} = \frac{1}{{\ln (1.02 + P_{{{\text{category}}}} )}}.$$

Besides, we employ OHEM strategy with threshold *t* = 0.7 and *N* = 100000 in the calculation of loss to tackle the problem of data imbalance existing between various categories, where *t* denotes the specific threshold to select hard pixels and *N* is the least pixels to be counted as hard pixels within each mini-batch [21].

Further, we fine-tune the model with Tversky loss after training the model with compound loss and aforementioned strategy. For our task of garbage segmentation, we place more emphasis on false negatives since missed detections are more severe mistakes than false positives. Therefore we adopt Tversky loss with *α* = 0.3, *β* = 0.7 to boost recall. The Tversky index over areas *A* and *B* can be described as

4 $$T(A,B) = \frac{{\left| {A \cap B} \right|}}{{\left| {A \cap B} \right| + \alpha \left| {A - B} \right| + \beta \left| {B - A} \right|}},$$

where *α* and *β* control the penalties for precision and recall.

**Fig. 7:**
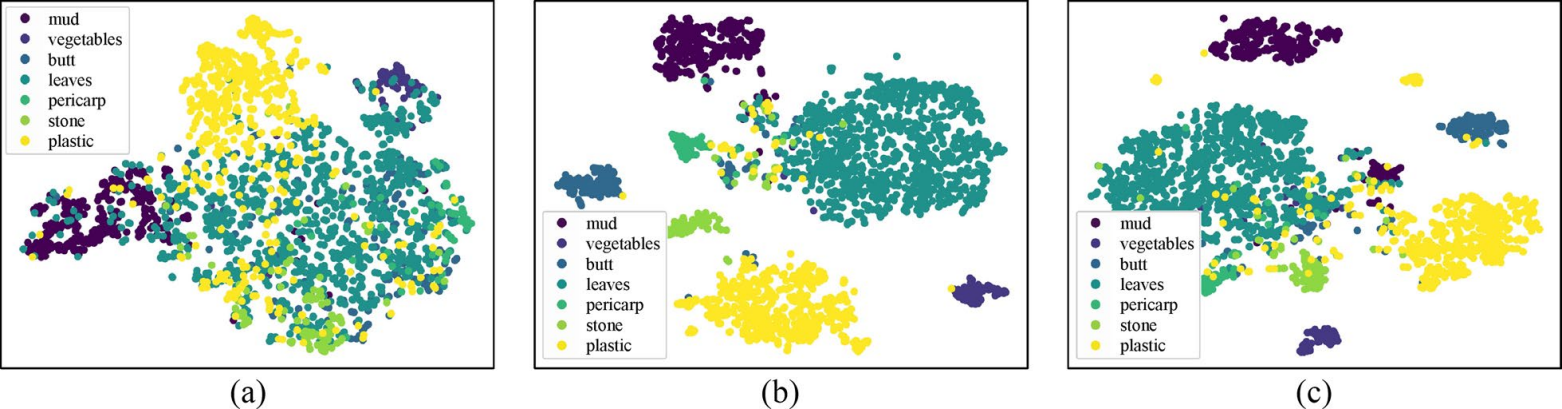
Distribution of feature maps trained with different losses. From left to right are results of **a** cross-entropy (CE) loss, **b** CE loss along with OHEM strategy, and **c** that fine-tuned with Tversky loss

Figure 7 demonstrates the distribution of feature maps trained with cross-entropy loss, OHEM strategy, and fine-tuned with Tversky loss, respectively. As can be seen from the distribution of feature maps, it is beneficial to apply the compound loss function as it yields a significant gain in making the model produce more distinguishable features.

## Experiments and discussion

### Garbage data sets

**Fig. 8:**
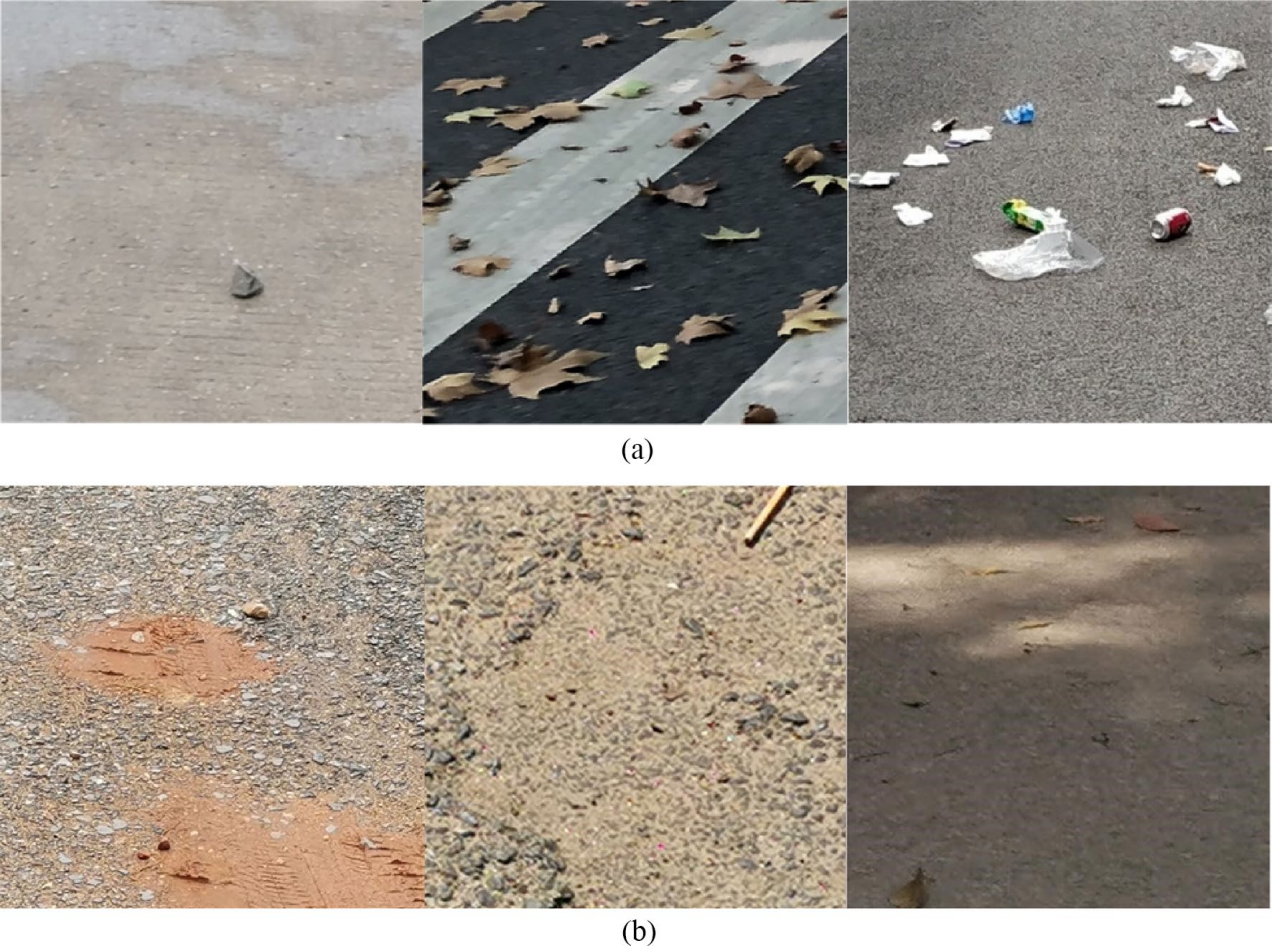
Samples of (**a**) road in campus (RdIC) and (**b**) road outside campus (RdOC) data sets

**Fig. 9:**
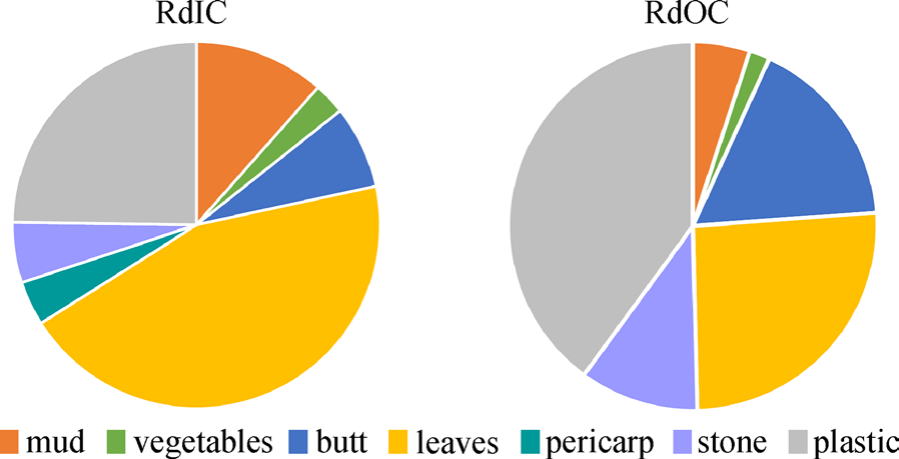
Image ratios of RdIC and RdOC data sets

We build two data sets to assess the performance of our model, one captured on the road in campus, called RdIC, targeting seven garbage categories (mud, vegetables, butt, leaves, pericarp, stone, plastic) and additional background, the other captured outside campus, called RdOC, including six garbage categories (the same as RdIC excluding pericarp category) and additional background. The two data sets are both cropped into a size of 512 × 512 pixels. RdIC contains 803 training and 245 validation/testing images, while RdOC contains 1496 training and 645 validation/testing images, respectively. Samples of different garbage categories of the two data sets are shown in Fig. 8. It can be seen that images of RdOC are taken from more diverse environments. Figure 9 shows image ratios of different categories in the two data sets, demonstrating that the image number of garbage under leaves and plastic occupies a major proportion in both scenes of our data sets.

### Implementation details

We employ mean intersection-over-union (mIoU) averaged across all categories to evaluate overall segmentation accuracy, defined as follows, while running time (ms), model size (M), and memory access cost (MAC) [29] are adopted to measure model complexity and implementing efficiency.

5 $${\text{MIoU}} = \frac{1}{k + 1}\sum\limits_{i = 0}^{k} {\frac{TP}{{FN + FP + TP}}} .$$

For fair comparison, all the experiments are conducted on the same hardware platform with an RTX 2070 GPU, and a mini-batch size of 6 is adopted in the entire method; moreover, the “poly” learning rate strategy is adopted with power 0.9, momentum 0.9, and weight decay 1⨯10^−4^. We adopt weighted cross-entropy loss for all state-of-the-art segmentation networks, and as part of our design, a compound loss is used for our model. We set initial learning rate as 5⨯10^−4^ for our model, ERFNet [10] and ENet [25], 2⨯10^−3^ for DeepLabs [14].

**Fig. 10:**
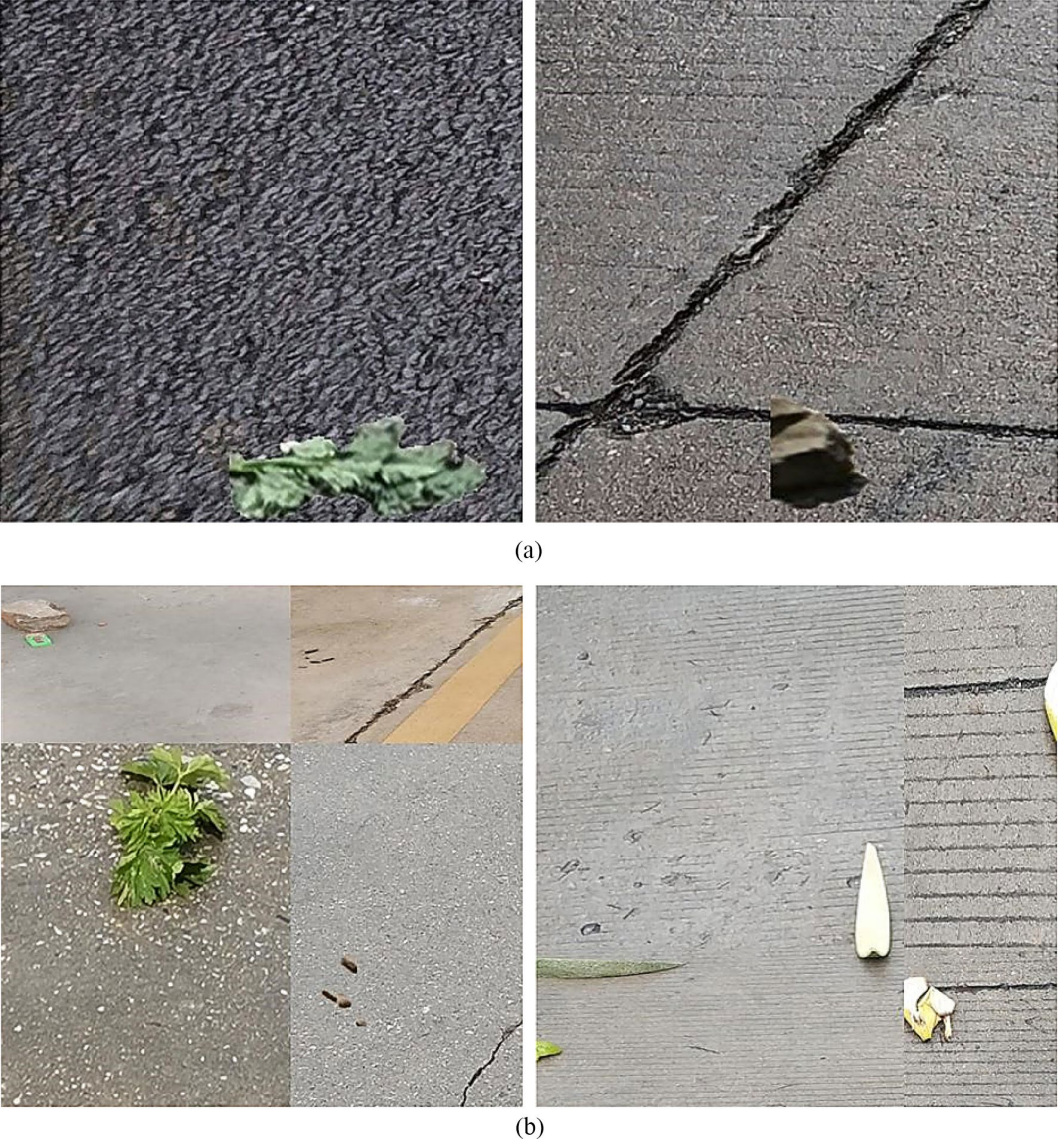
Samples of augmentation methods. **a** Lucid data dreaming. **b** Mosaic augmentation

Not only do we adopt conventional data augmentation strategies such as random flipping and Gaussian blur, but we also perform data augmentation via lucid data dreaming [30] and mosaic augmentation [31]. Lucid data dreaming is a simulation method that has achieved appealing results in video segmentation, which targets generating samples of objects from relatively rare categories under different backgrounds. After performing illumination change and deformation of the foreground target and dynamic background change, the foreground and background from another image are fused by Poisson matching [30]. Mosaic augmentation resizes and mixes four different training images into a single sample [31]. Unlike conventional data augmentation methods of pixel-wise, simulation and Mosaic methods can better increase the variability of the input images so that the model trained has higher robustness to the images obtained from different environments. In addition, the Mosaic method involves more different contexts in a training batch, which significantly reduce the demand for a large mini-batch size [31]. The samples of two augmentation methods are shown in Fig. 10.

### Comparison experiments

To show the advantages of our model, we select several state-of-the-art lightweight networks, including ERFNet [10], ENet [25] and DeepLab v3+ (Resnet50 and Resnet101 as the backbone) [14] as baselines. We also compare the proposed model results with the number of output channels of the decoder set as 32, 64, and 128, respectively.

**Fig. 11:**
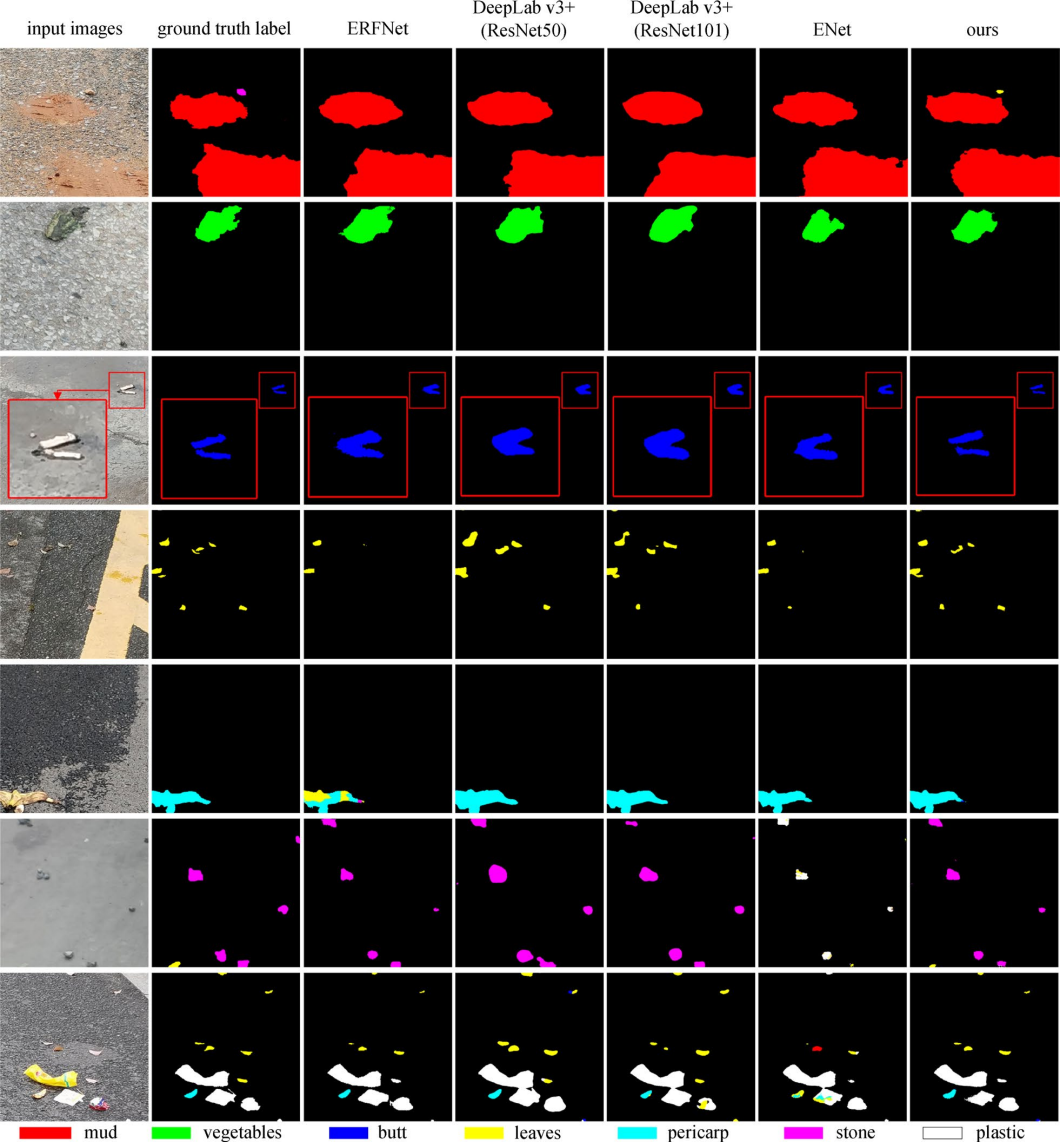
Examples of segmentation predictions on our garbage data sets. From left to right are input images, ground truth, segmentation outputs from ERFNet [10], DeepLab v3+ (ResNet50 as backbone), DeepLab v3+ (ResNet101 as backbone) [14], ENet [25] and our method

Tables 2 and 3 report comparison results. Among all the models we evaluated, our model achieves 87.8% and 67.5% category mIoU, respectively, where butt, leaves, and plastic categories produce the best accuracy on both data sets. Figure 11 shows the visualization of comparisons of the approaches above. As is demonstrated, our model not only segments and classifies garbage objects on the road with comparable accuracy but also produces more precise contours than the lightweight baselines, for example, in row 3 of Fig. 11, prediction results of the two butts are all overlapped except our model.

**Table 2:** Results of comparison experiments on RdIC data set

Model	IoU							mIoU
	Mud	Vegetables	Butt	Leaves	Pericap	Stone	Plastic	
ERFNet	0.8516	0.9621	0.6168	0.6961	0.7961	0.2625	0.9219	0.7623
DeepLab v3+ (ResNet50)	0.7947	0.9446	0.5928	0.7390	0.8420	0.4683	0.9033	0.7845
DeepLab v3+ (ResNet101)	0.8574	0.9459	0.5877	0.7476	0.8536	0.6056	0.9054	0.8119
ENet	0.8721	**0.9650**	0.6940	0.7981	0.8342	0.5167	0.9312	0.8258
Ours	**0.8780**	0.9602	**0.7549**	**0.8542**	**0.9314**	**0.7017**	**0.9469**	**0.8779**

The bold represents the maximum value in the same category of experimental data, and the underline represents the second largest value

**Table 3:** Results of comparision experiments on RdOC data set

Model	IoU						mIoU
	Mud	Vegetables	Butt	Leaves	Stone	Plastic	
ERFNet	0.6329	0.6137	0.4921	0.5973	0.3040	0.7876	0.6304
DeepLab v3+ (ResNet50)	0.6518	0.7117	0.5087	0.5826	0.2554	0.7356	0.6328
DeepLab v3+ (ResNet101)	0.5630	**0.7187**	0.4940	0.6200	**0.4832**	0.7751	0.6623
ENet	0.6120	0.5992	0.3928	0.5401	0.0017	0.7538	0.5550
Ours	**0.6596**	0.6351	**0.5297**	**0.6483**	0.4548	**0.8083**	**0.6748**

The bold represents the maximum value in the same category of experimental data, and the underline represents the second largest value

**Fig. 12:**
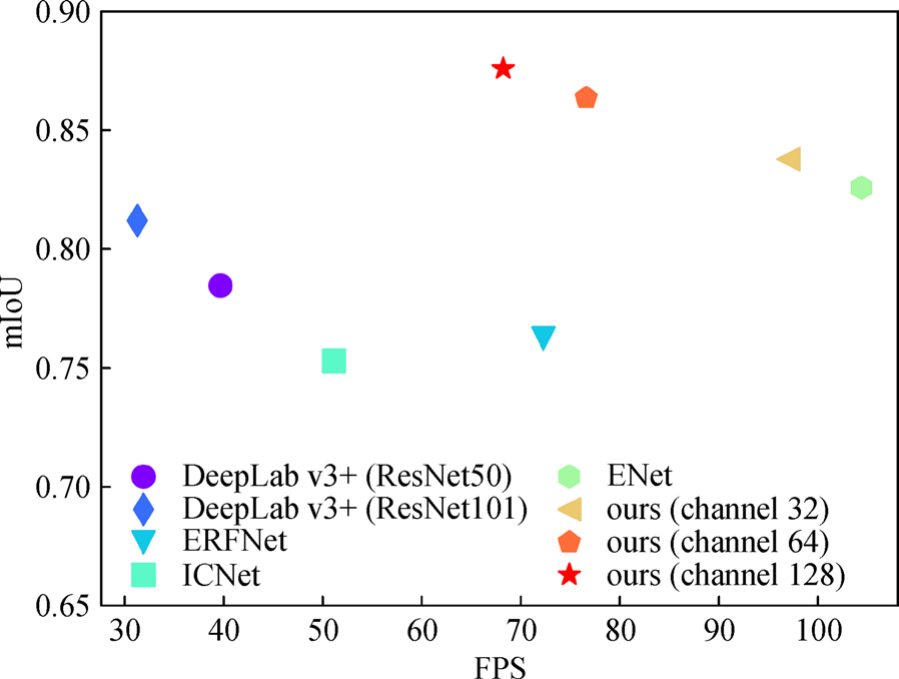
Measurement of accuracy and speed of segmentation models

When efficiency is considered, the proposed method yields competitive results in terms of speed and accuracy trade-offs. As demonstrated in Fig. 12 and Table 4, our model with 128 channels of the decoder is nearly twice as fast as DeepLab v3+ (ResNet101 as the backbone), in the meantime obtains comparable segmentation results with approximately 20 times fewer parameters. Although ENet is more efficient in computation complexity, the segmentation result is less appealing, with an accuracy drop of 5% and 12% for two data sets compared to our model.

**Table 4:** Complexity and accuracy of models

Model	Model complexity			mIoU	
	Params/M	MAC/GMac	Time/ms	RdIC	RdOC
ERFNet	2.06	13.38	13.8	0.7623	0.6304
DeepLab v3+ (ResNet50)	40.35	69.40	25.1	0.7845	0.6328
DeepLab v3+ (ResNet101)	59.34	88.87	31.8	0.8119	0.6623
ENet	0.35	2.03	9.6	0.8258	0.5550
Ours (channel 32)	2.18	11.38	10.3	0.8377	0.6311
Ours (channel 64)	2.32	13.60	12.1	0.8635	0.6478
Ours (channel 128)	2.72	15.23	14.6	0.8779	0.6748

Further, to demonstrate the effectiveness of the proposed mechanism, we compare the semantic segmentation results with the original ERFNet model over the Cityscape data set. Both methods are trained using fine data only. Table 5 shows the comparison results, where our method produces better IoU over 17 out of the 19 categories and yields 70.32% mIoU, which is 2% higher than that of the original ERFNet.

**Table 5:** Comparison results on Cityscapes data set

Method	IoU					
ERFNet	Roa	Sid	Bui	Wal	Fen	Pol
	0.9645	**0.7825**	0.9020	0.3928	0.5111	0.5710
	Sky	Ped	Rid	Car	Tru	Bus
	0.9358	0.7507	0.5179	0.9257	0.6373	0.7113
Ours	Roa	Sid	Bui	Wal	Fen	Pol
	**0.9734**	0.7694	**0.9075**	**0.4766**	**0.5116**	**0.5866**
	Sky	Ped	Rid	Car	Tru	Bus
	**0.9387**	**0.7630**	**0.5353**	**0.9290**	**0.6889**	**0.7552**

Method	IoU				mIoU	
ERFNet	TLi	TSi	Veg	Ter	0.6821	
	0.6103	0.6962	0.9109	**0.5997**		
	Tra	Mot	Bic			
	0.4907	0.3528	0.6969			
Ours	TLi	TSi	Veg	Ter	**0.7032**	
	**0.6278**	**0.7176**	**0.9123**	0.5865		
	Tra	Mot	Bic			
	**0.5909**	**0.3890**	**0.7008**			

The bold represents the maximum value in the same category of experimental data

### Ablation experiments

To demonstrate the effectiveness of each component in the proposed method, we designed the following experiments as presented in Table 6, evaluating feature pyramid attention module, SG module, feature concatenation path, OHEM strategy, and Tversky fine-tune loss and two data augmentation method for segmentation. Except for the component evaluated, all the experiments share the identical experimental setups.

**Table 6:** Ablation experiments setups

Model	Structure description	Training strategy
w/o FPA	Without FPA module	Proposed
w/o SG	Without SG module	Proposed
w/o concat	Without concatenation path between encoder and decoder	Proposed
w/o compound loss	Proposed model	Only weighted cross-entropy loss
w/o augment	Proposed model	Without mosaic and lucid data dreaming

Tables 7 and 8 show the results of ablation experiments. Adopting a modified FPA module and a concatenation path between encoder and decoder can improve the overall segmentation accuracy at the cost of only a little reduction of the network operation speed. Regarding strategies that do not give rise in any computation cost in the prediction stage, the model trained with the proposed loss function and adopting the proposed data augmentation method produces better performance than that with common weighted cross-entropy loss and conventional augmentation. It is worth mentioning that the strategies work more significantly for those categories with relatively rare proportions. For example, without mosaic and lucid data dreaming augmentation, the IoU value of the stone category drops 26% and 7% on two data sets, and without compound loss functions, the IoU value of the butt category is both 8% lower. Besides, as demonstrated above, the performance gap between models with and without SG module is less significant under the circumstance of using abundant training samples. Introducing the SG module is more effective in training with limited samples, which is detailed in the following section.

**Table 7:** Results of ablation experiments on RdIC data set

Model	IoU							mIoU
	Mud	Vegetables	Butt	Leaves	Pericarp	Stone	Plastic	
w/o FPA	**0.8919**	**0.9770**	0.7092	0.8419	0.8915	0.4340	0.9427	0.8355
w/o SG	0.8689	0.9585	**0.7588**	**0.8603**	**0.9359**	0.5804	**0.9496**	0.8635
w/o concat	0.8564	0.9712	0.7134	0.8354	0.9142	0.6319	0.9415	0.8574
w/o compound loss	0.8566	0.9548	0.6758	0.8266	0.9126	0.5816	0.9310	0.8418
w/o augment	0.8730	0.9723	0.7516	0.8214	0.9033	0.4362	0.9380	0.8363
Proposed	0.8780	0.9602	0.7549	0.8542	0.9314	**0.7017**	0.9469	**0.8779**

The bold represents the maximum value in the same category of experimental data, and the underline represents the second largest value

**Table 8:** Results of ablation experiments on RdOC data set

Model	IoU						mIoU
	Mud	Vegetables	Butt	Leaves	Stone	Plastic	
w/o FPA	0.6227	0.6129	0.4642	0.6389	0.4649	0.8108	0.6573
w/o SG	0.6553	0.5947	**0.5977**	**0.6488**	0.4239	**0.8200**	**0.6755**
w/o concat	0.5935	0.6259	0.4852	0.6168	**0.4756**	0.7527	0.6478
w/o compound loss	0.6072	**0.6740**	0.4440	0.6415	0.3854	0.8069	0.6492
w/o augment	0.5064	0.5646	0.4396	0.5753	0.3889	0.7826	0.6059
Proposed	**0.6596**	0.6351	0.5297	0.6483	0.4548	0.8083	0.6748

The bold represents the maximum value in the same category of experimental data, and the underline represents the second largest value

### Experiments on subset data sets

**Fig. 13:**
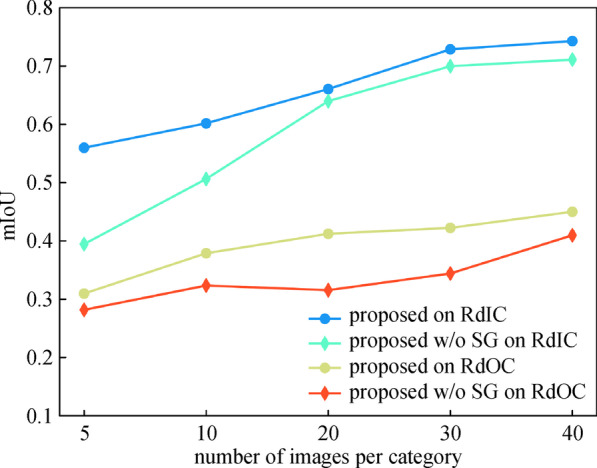
mIoU evaluation of proposed model with and w/o SG module

**Fig. 14:**
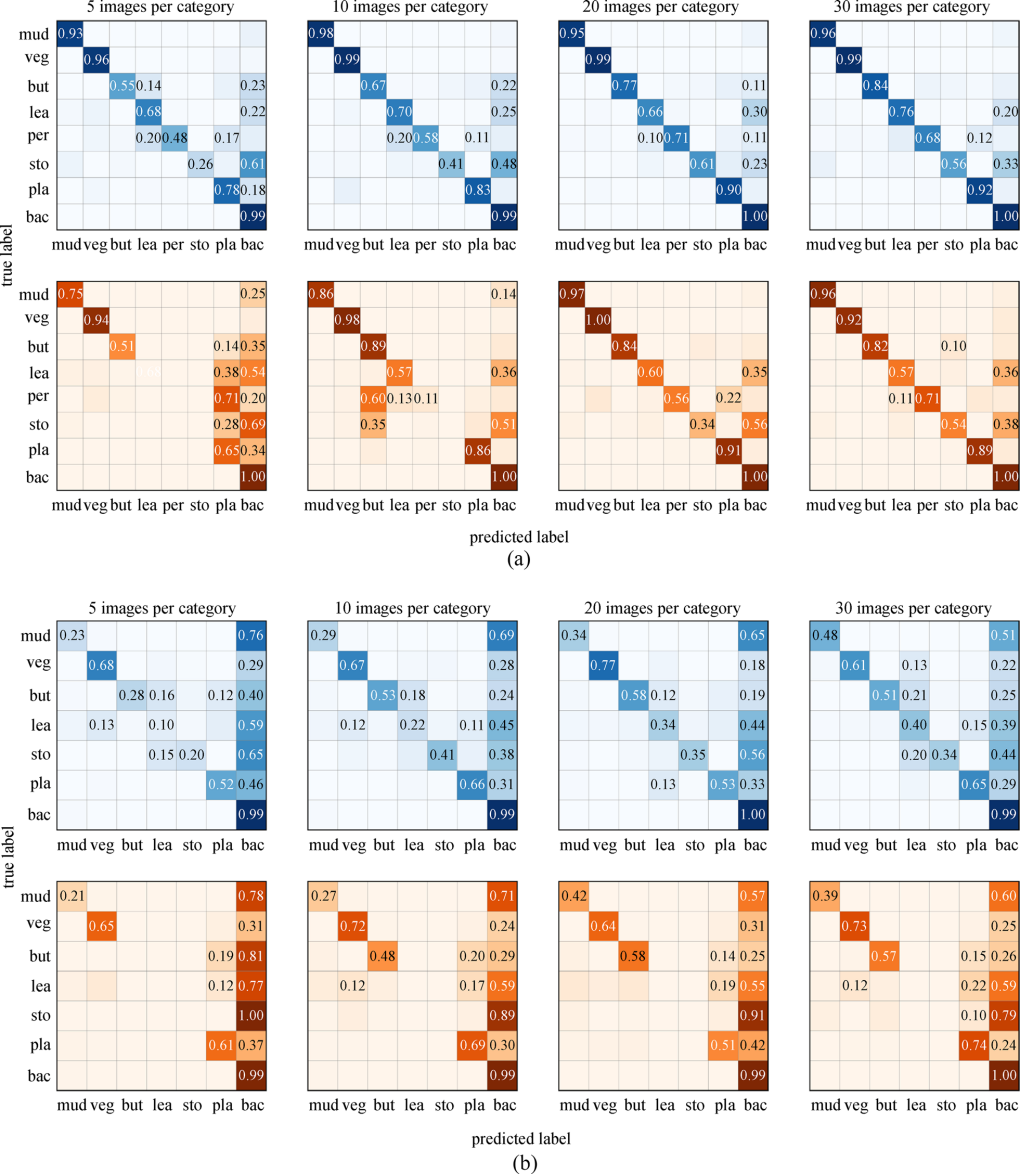
Confusion matrix of predictions on two data sets. From up to down are results of proposed model and model without SG module. **a** is the results of models trained on RdIC data set. **b** is for models trained on RdOC data set. veg: vegetables, but: butt, lea: leaves, sto: stone, pla: plastic, bac: background

To prove the superiority of our model in tackling the shortage of training samples, we perform experiments on subsets of the training set. We randomly select 5, 10, 15, 20, 30, 40 images respectively for each garbage category from training set. Figures 13 and 14 list the comparison results via mIoU and confusion matrix.

Figure 13 demonstrates that the model with SG module prevails that without SG module on overall mIoU rate with less than 20 training images for each category. It can be seen from the confusion matrixes in Fig. 14 that when trained with over 20 samples, although the mIoU gain of introducing SG module does not outstand by a large margin, the model with SG module yet yields more balanced results in terms of recall rate of each category. When the training set is inadequate, a model without an SG module tends to go to extremes to ensure the recall rate of easy category, giving rise to false negatives of those hard categories such as butt, leaves, and stones. The model with an SG module, in contrast, generates predictions not only based on class activation of the feature maps but also the similarity distance between the prototype feature vector and each pixel in all positions of the input image, thus involving the SG module forces the model to consider every category in an equal way. Our model with an SG module can perform segmentation from only a few annotated images and produces consistently category-balanced results, and learns effectively from an abundant training set.

### Experiments in practical application scenarios

To evaluate the effectiveness of our model applied in practical application scenarios, we collect a practical data set of images with the size of 2432 × 896 pixels, captured by the industrial camera, which simulates the practical situation. The practical data set contains 115 training and 50 validation/testing images. After pretrained with the RdOC data set and fine-tuned with the practical training samples, our model reaches mIoU of 0.7113 on the test set of practical images, showing the promising generalization ability of the model.

**Fig. 15:**
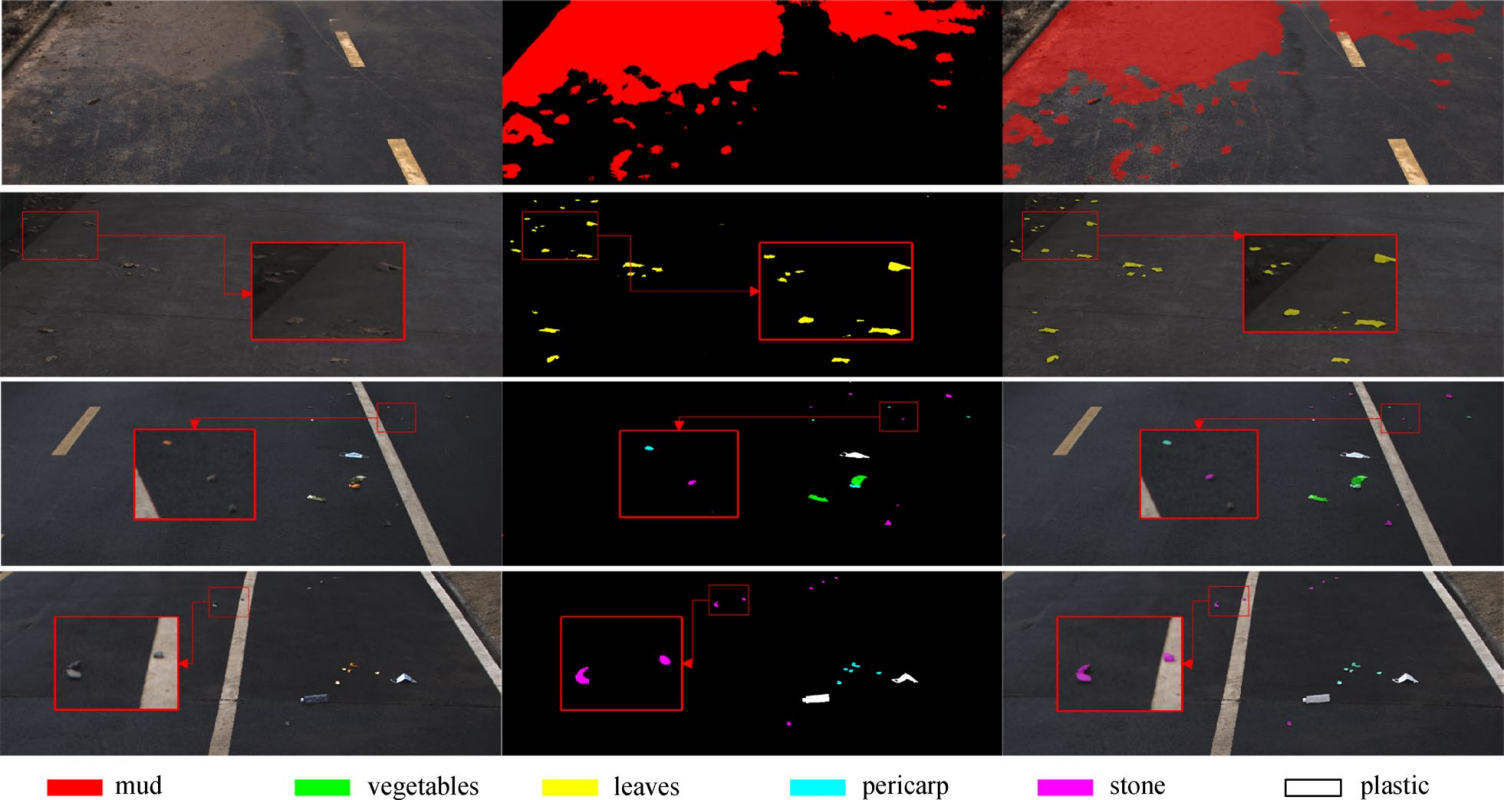
Qualitative samples of some representative road scene images. From left to right are input images, segmentation outputs, and visual demonstration of results

Segmentation results of some representative samples of road scene are summarized in Fig. 15. The proposed method performs well on the coverage and density measurement for the mud category; also, the method produces acceptable segmentation for small and scattered objects, such as leaves, pericarp, and stones, with promising classification accuracy. An example of a missed object is shown in row 3 of Fig. 15, possibly due to that the feature of the stone is undistinguished in terms of road background. Besides, it only takes 0.11 s for processing each image, which meets the actual technical requirements for our task.

## Conclusions

We present an efficient method for the task of real-time garbage segmentation under road scene. Our model is preferable for the task for several advantages. First, it is efficient in terms of speed and accuracy trade-offs. Our model adopts a lightweight structure, along with an FPA module and long-range skip connection, which contribute to accuracy without adding too much burden on computation budgets. Besides, we alleviate the imbalance issue by improving training strategies, including introducing OHEM strategy and adopting various data augmentations. Second, the method shows a promising perspective in landing to practical scenarios. By adding an SG module to the decoder branch, the model can produce acceptable segmentation results with only a limited number of annotated images, and thus the difficulty of training the model is decreased by a large margin. Experimental results show that our method achieves overall mIoU of 0.87 and 0.67 respectively on two garbage data sets we built and can produce acceptable category-balanced segmentation with less than 20 annotated samples for each category. Also, the method reaches 65 FPS for image size 512 × 512 in an RTX 2070 GPU and 9 FPS for image size 2432 × 896, which indicates inference time of our model meets the actual technical requirements of road sweepers in street cleaning work.

In the future, we would continue to tackle the challenges arising in applying our method in the practical use of intelligent control of road-sweepers. First, our samples only include road scenes with good lighting conditions; in the future, we would continue to study specific road scenes (rainy scenes, or night scenes, for example) and improve the robustness of our method to poor lighting conditions. Second, our experiments were all conducted on a GPU platform of a desktop computer; more efforts would be made to optimize model deployment and model acceleration in an embedded system to meet the practical requirements.
